# Dissection of niche competition between introduced and indigenous arbuscular mycorrhizal fungi with respect to soybean yield responses

**DOI:** 10.1038/s41598-018-25701-4

**Published:** 2018-05-09

**Authors:** Rieko Niwa, Takuya Koyama, Takumi Sato, Katsuki Adachi, Keitaro Tawaraya, Shusei Sato, Hideki Hirakawa, Shigenobu Yoshida, Tatsuhiro Ezawa

**Affiliations:** 1 Central Region Agricultural Research Center, National Agriculture and Food Research Organization (NARO), 2-1-18 Kannondai, Tsukuba, 305-8666 Japan; 2 Kyushu Okinawa Agricultural Research Center, NARO, 6651-2 Miyakonojo, Miyazaki, 885-0091 Japan; 3 Faculty of Agriculture, Yamagata University, Tsuruoka, 997-8555 Japan; 4 Graduate School of Life Sciences, Tohoku University, Sendai, 980-8577 Japan; 5 Kazusa DNA Research Institute, Kisarazu, 292-0818 Japan; 6 Graduate School of Agriculture, Hokkaido University, Sapporo, 060-8589 Japan; 7 Present Address: Institute for Horticultural Plant Breeding, 2-5-1 Kamishiki, Matsudo, Chiba 270-2221 Japan; 8 Present Address: School of Agriculture, Utsunomiya University, 350 Mine-machi, Utsunomiya, Tochigi 321-8505 Japan

## Abstract

Arbuscular mycorrhizal (AM) fungi associate with most land plants and deliver phosphorus to the host. Identification of biotic/abiotic factors that determine crop responses to AM fungal inoculation is an essential step for successful application of the fungi in sustainable agriculture. We conducted three field trials on soybean with a commercial inoculum and developed a new molecular tool to dissect interactions between the inoculum and indigenous fungi on the MiSeq sequencing platform. Regression analysis indicated that sequence read abundance of the inoculum fungus was the most significant factor that determined soybean yield responses to the inoculation, suggesting that dominance of the inoculum fungus is a necessary condition for positive yield responses. Agricultural practices (fallow/cropping in the previous year) greatly affected the colonization levels (i.e. read abundances) of the inoculum fungus via altering the propagule density of indigenous AM fungi. Analysis of niche competition revealed that the inoculum fungus competed mainly with the indigenous fungi that are commonly distributed in the trial sites, probably because their life-history strategy is the same as that of the inoculum fungus. In conclusion, we provide a new framework for evaluating the significance of environmental factors towards successful application of AM fungi in agriculture.

## Introduction

Arbuscular mycorrhizal (AM) fungi that belong to the subphylum Glomeromycotina form mutualistic associations with about 80% of land plants, including important agricultural crops, and deliver mineral nutrients, especially phosphate, to the host^1^. Potential of AM fungi in the improvement of crop yield, therefore, has been studied in the context of sustainable agriculture^2–5^.

Soybean (*Glycine max* (L.) Merrill.) is the most widely grown leguminous crop that provides an important source of protein and oil. Recently, increases in yield by inoculation of AM fungi in field trials have been reported in soybean^6–8^. A meta-analysis of the published studies on 8 legume crops revealed that, although ‘strict’ non-mycorrhizal control was absent under the field conditions, the changes in yield by AM fungal inoculation varied from −4% to +24% and was only +9% on average, which was marginal compared with those in the pot experiments (45% increase in average)^9^. Accordingly, identification of biotic and abiotic factors that determine soybean responses to AM fungal inoculation in the field is an essential step for successful application of AM fungi in agricultural production.

One crucial biotic factor is indigenous AM fungi; they are well adapted to the local environment^10^ and thus competitive with introduced (inoculum) fungi^4^. Various agricultural practices/management alter indigenous AM fungal community qualitatively and quantitatively. The long-term application of chemical fertilizer^11,12^ and tillage^13^ have a significant impact on the community compositions. Organic farming increases species richness of the fungi, compared with that in conventional agriculture^14,15^. Cropping of AM fungal host plants improves growth of succeeding crops via increasing indigenous AM fungal population^16^. It has been suggested that these factors affect the effectiveness of AM fungal inoculation^17^. For example, long-term fallow decreases the propagule density of indigenous AM fungi and increases the responsiveness of leek to AM fungal inoculation^18^, suggesting that the inoculation of the fungi is more effective under conditions where population of indigenous fungi is smaller or they perform poorer. Little is known, however, about how agricultural practices/management affect the responsiveness of plants to the inoculation via altering the interactions between indigenous and introduced AM fungi.

Specific molecular markers for tracking introduced AM fungi in the field have been developed. Two strains of non-native *Funneliformis mosseae* were successfully traced and discriminated from native strains of the species by PCR-RFLP targeting the large subunit (LSU) ribosomal RNA gene (rDNA)^2^. In *Rhizophagus irregularis* genotype-specific markers targeting simple sequence repeats, a nuclear gene intron, and mitochondrial LSU rDNA introns have also been developed^19^. To dissect the interactions between introduced and indigenous fungi, however, not only particular fungal strains but also indigenous AM fungi should simultaneously be detected on the same platform. The rDNA transcription unit, including the small subunit (SSU), internal transcribed spacer (ITS), and LSU, has most commonly been employed for dissecting AM fungal community. In this approach, taxonomic resolution of the fungi greatly depends on the combination of the target region of rDNA and sequencing platform. On the Sanger (clone library/random sequencing) and Roche 454 platforms, sequencing of the SSU rDNA, followed by the assignment of the sequences to the virtual taxa in the Maarj*AM* database^20,21^, is the most common approach^22^. Recently, Schlaeppi *et al*.^23^ demonstrated that introduced AM fungi were traceable in the field via sequencing a 1.5-kbp region spanning the SSU rDNA, ITS, and LSU rDNA on the medium-throughput sequencing platform PacBio. Whereas Illumina MiSeq generates short (300 bp) but a massive number (up to 50 M) of paired-end reads, which is a great advantage for community ecology, and in fact, the platform has become standard in bacterial ecology^24,25^. To take this advantage in AM fungal ecology, we chose the LSU rDNA as a target for MiSeq sequencing. In AM fungi, the sequence variations in the LSU rDNA provide high-resolution taxonomic information^26^ and have widely been employed in the ecology^11,27–30^. Within the LSU rDNA, in particular, the divergent domain 2 (D2) that is less than 400 bp in length is short enough to cover by MiSeq 300-bp paired-end sequencing and provides sufficient taxonomic resolution^31^.

Here, we provide a framework for identifying biotic and abiotic factors that determine the responsiveness of soybean to AM fungal inoculation in the field through tracking an inoculum AM fungus in indigenous community, in which the following two hypotheses were addressed; (i) the dominance of inoculum fungus in the host roots is a necessary condition for positive yield responses, and (ii) the propagule density of indigenous AM fungi largely determines the extent of the colonization of inoculum fungus. To test these hypotheses, we developed molecular tools adapted to the MiSeq sequencing platform, including a database for taxonomic assignment and a data processing pipeline driven via an open web interface, for high-throughput community analysis of the fungi.

## Materials and Methods

### Fungal inoculum

An AM fungal inoculum *Glomus* sp. strain R-10 (R-10) was purchased from Idemitsu Kosan Co., Ltd., Tokyo. The inoculum consists of spores and root fragments of R-10 with a crystalline-silica carrier. For the control treatments, we obtained the carrier that was free of the propagule (i.e. unprocessed carrier) from the manufacturer. The most probable number (MPN) of the inoculum was 14 propagules g^−1^, which was determined prior to the field trials as described in Supplementary Methods S1. To define a DNA tracking marker for R-10, the inoculum was mixed with sterilized soil at a rate of 50 g kg^−1^ soil, and *Allium fistulosum* cv. Motokura was grown in nursery pots in a greenhouse. After 42 days, the roots were harvested, freeze-dried, and stored at −30 °C for DNA extraction and sequencing.

### Field trials and sampling

Three trials were designed in adjacent field sites in Kyushu Okinawa Agricultural Research Center, National Agricultural and Food Research Organization, Miyakonojo, Miyazaki, Japan (31°45′05′′N 131°00′46′′E). In the preceding year 2014, trials 1 and 2 were bare fallowed (T1_BF and T2_BF), whereas palisade grass *Brachiaria brizantha* (Hochst. ex A. Rich.) cv. MG5 was grown from July to Sep in trial 3 (T3_PG). The fields for T1_BF and T2_BF were periodically plowed to control weeds in 2014. Details of the environmental conditions are described in Supplementary Methods S2. In 2015 soil samples were collected from each replicated block (*n* = 4) prior to fertilizer application to determine the MPN of AM fungal propagule (Supplementary Methods S1). All these trial sites have a history of soybean cultivation, and thus nitrogen-fixing nodules are formed by native rhizobia (i.e. without rhizobial inoculation). Phosphorus (P) fertilizer was applied at three different levels, 0 (P0), 50 (P50), and 100 (P100) kg P_2_O_5_ ha^−1^, in T1_BF and at two different levels, 0 (P0) and 100 (P100) kg P_2_O_5_ ha^−1^, in T2_BF and T3_PG. Nitrogen (N) and potassium (K) fertilizers were applied at 40 kg N ha^−1^ and 120 kg K_2_O ha^−1^, respectively, in all trials. Five grams of either the inoculum or the propagule-free carrier was placed at a depth of 80 mm and buried, and then three seeds of *Glycine max* (L.) Merrill. cv. Fukuyutaka were sown on 27 and 28 July in T1_BF and on 24 July in T2_BF and T3_PG and thinned to two plants after the first trifoliate appeared. The replicate plot size in Trial 1 (T1_BF) was 3.9 × 4.4 m, and that in Trial 2 and 3 (T2_BF and T3_PG) was 3.9 × 2.8 m, in which six rows were arranged with distance of 0.65 m between rows (0.2 m between hills). Weed in the plots were controlled manually. Intertillage and ridging were conducted in the middle of Aug. The treatments were arranged in the randomized complete block design.

At the time of flowering (Aug 31–Sep 4), roots and root-zone soils (20 × 20-cm square, 25 cm in depth) were collected from four plants in each plot and combined. After gentle washing of the roots with tap water, about 1 g of subsamples (lateral roots attached to the tap root) were collected in the middle of the tap root, cut into 1-cm segments, randomized in water, blotted on a paper towel, freeze-dried, and stored at −30 °C for DNA extraction. The soil samples were dried in greenhouse, passed through a 2-mm stainless sieve, and stored at room temperature for chemical analysis. The above ground part was harvested on 11 Nov in T1_BF and on 24 Nov in T2_BF and T3_PG from 24 plants grown within 1.56 m^2^ (1.3 × 1.2 m) in each plot, and grain yield of 15% moisture content was recorded after drying at 80 °C for 72 h. For measuring total biomass and P concentration of the above ground part, another four plants were harvested in each plot, combined, dried at 80 °C for 72 h, weighed, ground with a mill, and stored in a desiccator.

### Soil and plant analyses

Soil pH (H_2_O) was measured at a 1: 2.5 soil: water ratio (w/v) using an electrode after shaking for 1 h at 160 rpm. Total carbon (C) and N were analyzed with a CNS analyzer. Available phosphorus was extracted according to Truog (1930)^32^ with a modification in the extraction buffer^11^ and measured colorimetrically. Exchangeable calcium (Ca), magnesium (Mg), and K were displaced with 1 M ammonium acetate and measured with atomic absorption spectroscopy (Ca and Mg) and flame photometry (K). Cation exchange capacity was determined by summation of exchangeable base cation and exchangeable acidity. Nitrate and ammonia concentrations were determined by the hydrazine reduction and indophenol methods, respectively. Phosphate absorption coefficients were measured by the ammonium phosphate methods. The ground plant samples were digested with a mixture of nitric acid-perchloric acid according to Miller (1998)^33^, and P concentration in the digests was determined by the vanadomolybdate-yellow assay^34^.

### DNA extraction and PCR amplification

The freeze-dried root samples were ground in 3-mL tubes with a metal cone at 3,000 rpm for 45 s at room temperature using Multi-Beads Shocker (Yasui Kikai, Osaka), and DNA was extracted from the ground samples (10–20 mg) with DNeasy Plant Mini Kit (Qiagen, Tokyo) according to the manufacturer’s instructions, and stored at −30 °C.

**Figure 1:**
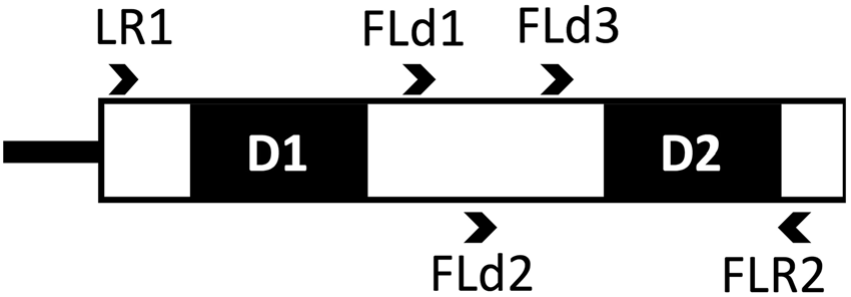
Relative locations and directions of PCR primers for amplification of the large subunit ribosomal RNA gene. Three forward primers, FLd1, FLd2 and FLd3, were designed in the region between the divergent domains 1 (D1) and 2 (D2).

Three mixed PCR primers, FLd1, FLd2, and FLd3, were designed in the conserved region between the D1 and D2 in the LSU rDNA (Fig. 1 and Supplementary Table S1), and their performance was assessed prior to the main experiment by comparing with the eukaryote-universal primer LR1^35^ in combination with the fungi-specific reverse primer FLR2^36^ (Supplementary Methods S3). In the main experiment the region was amplified in a 25-µL reaction mixture of Expand High-Fidelity PLUS PCR System (Roche Diagnostics, Tokyo), 0.5 µM of the primers, and five different amounts of DNA template (0.01, 0.02, 0.1, 0.5, and 1.0 µL per 25 µL for each sample) using C1000 Touch Thermal Cycler (BIO-RAD, Tokyo) with the following program: initial denaturation at 94 °C for 2 min, followed by 30 cycles of denaturation at 94 °C for 15 s, annealing at 48 °C, polymerization at 72 °C for 1 min, and final extension at 72 °C for 10 min. A preliminary experiment showed that the relative abundances of different AM fungal sequences in the PCR products raised from different amounts of DNA template differed from each other. Accordingly, all PCR products obtained from the five different amounts of DNA template were combined, purified with Agencourt AMPure XP PCR Purification System (Beckman Coulter, Tokyo), and subjected to sequencing.

### Sequencing and data processing

The first PCR products were subjected to the 2nd PCR to attach dual indices and Illumina sequencing adaptors using Nextera XT Index Kit v2 (Illumina, Tokyo) according to the 16 S Metagenomic Sequencing Library Preparation protocol (https://support.illumina.com/downloads/16s_metagenomic_sequencing_library_preparation.html). The libraries were pooled, adjusted to 4 nM DNA, denatured with NaOH, and then diluted with the hybridization buffer to the final concentration of 6 pM DNA, and 300-bp paired-end sequencing was carried out by the Illumina MiSeq platform using MiSeq Reagent Kit v3 (600 Cycles) (Illumina K.K., Tokyo).

The nucleotides with a quality value (QV) <30 in the 3′ terminal and the adapter-index sequence in the 5′ terminal were trimmed from the MiSeq reads by PRINSEQ v0.20.4^37^, and those shorter than 200 bp were excluded. After the quality filtering, an overlap fragment of the 300-bp-paired reads (read 1 and read 2) were constructed by using COPE v1.1.3^38^ with the minimum and maximum overlap lengths of 10 and 300 nt, respectively. The merged sequence reads were subjected to BLASTN searches against reference sequences (database) composed of 412 operational taxonomic units (OTUs) of glomeromycotinan (AM) fungi defined in this study (Supplementary Methods S4, Table S5, and Fig. S1) and 82,208 sequences of non-glomeromycotinan fungi obtained from Ribosomal Database Project^39^, in which sequence reads similar to an AM fungal OTU and those similar to a non-glomeromycotinan species were assigned to the OTU/species with different criteria as follows. For the reads similar to AM fungal OTUs, only those that met the criteria of E-value ≤ −100, ≥95% nt identity, and alignment length ≥330 bp with one of the AM fungal OTUs were assigned to the OTU. Whereas, for the reads similar to non-glomeromycotinan sequences, only those that met the criteria of E-value ≤ −100, ≥95% nt identity, and alignment length ≥220 bp with one of the 82,208 sequences were assigned to the species. All these analyses were executed in the open web interface “Arbuscular mycorrhizal fungi classification pipeline” (http://amfungi.kazusa.or.jp) constructed in this study.

### Statistical analysis

All statistical analyses were performed with R 3.2.3^40^. Two-way analysis of variance (ANOVA) with random effects for block differences were applied to evaluate the effects of the inoculation and P fertilizer application on shoot P concentration and grain yield. Vegan package for R^41^ was employed for β-diversity analysis with Bray-Curtis similarity index (read-abundance data based index) and for permutation multivariate analysis of variance (PERMANOVA) in which Bray-Curtis index was used as a measure of similarity (9999 permutations). In regression analyses all OTUs obtained by sequencing of the inoculum fungus R-10 were combined as ‘R-10-type OTUs’, in which ‘read abundance of the R-10-type OTUs’ represents the sum of the sequence reads assigned to the OTUs. Simple linear regression models were applied to analyze correlations between read abundances of the R-10-type OTUs (log-transformed) and MPNs of indigenous AM fungal propagule (log-transformed) and between the read abundances and soybean yield responses to the inoculation. The yield responses were calculated by the equation (1) as follows:

1 $$\begin{array}{c}{\rm{Yield}}\,{\rm{response}}\,{\rm{to}}\,{\rm{inoculation}}\\ =({\rm{Yield}}\,{\rm{in}}\,{\rm{inoculated}}\,{\rm{plot}}-{\rm{Mean}}\,{\rm{yield}}\,{\rm{in}}\,{\rm{control}}\,{\rm{plots}})/{\rm{Mean}}\,{\rm{yield}}\,{\rm{in}}\,{\rm{control}}\,{\rm{plots}}\end{array}$$

The multiple linear regression model was applied to evaluate to the effects of the read abundance of R-10-type OTUs and the environmental factors (Supplementary Table S6) on the yield responses to the inoculation. The best model was selected with reference to Akaike’s information criterion (AIC)^42^ calculated by the stepwise method using MASS package in R. The logistic regression model with random effects for block differences was applied to evaluate the effect of environmental factors on the read abundance of R-10-type OTUs using glmmML package in R^43^. The best model was selected with reference to AIC calculated using MuMIn package.

To dissect competition between the introduced (inoculum) and indigenous AM fungi, the following two indices were employed. ‘Commonness’ that is the index referred to as niche breadth in Levins (2013)^44^ and Pandit *et al*.^45^ was employed to evaluate their habitat specialization and calculated for each indigenous OTU by the equations (2 and 3) as follows:

2 $${\rm{Commonness}}=1/{\sum }_{(i=0)}^{n}{P}_{ij}^{2}$$

3 $${P}_{ij}=\frac{{\rm{Mean}}\,{\rm{read}}\,{\rm{number}}\,{\rm{of}}\,\mathrm{OTU}\,\,j\,{\rm{in}}\,{\rm{trial}}\,i}{{\rm{Sum}}\,{\rm{of}}\,{\rm{mean}}\,{\rm{read}}\,{\rm{numbers}}\,{\rm{of}}\,{\rm{OTU}}\,j\,{\rm{in}}\,{\rm{each}}\,{\rm{tiral}}}$$

where only the read number data in the uninoculated control were used to compute. OTUs with a higher value of commonness distribute more evenly across the habitats and thus are considered to be habitat generalists^45^. Rare OTUs of which the mean relative abundance was less than 0.2% of total read were not considered in this analysis. Robustness of each indigenous OTU against the introduction of R-10 fungus was defined as a ratio of the mean read abundance in the inoculated plots to that in the control plots in each trial.

### Data availability

All data used in this study are disclosed in the paper and supplementary information files. The raw data are available from the corresponding author on reasonable request.

## Results

### Validation of new primer for MiSeq platform

Approximately 350–450 bp fragments of the D2 were successfully amplified from the DNA extract of the maize test sample both with FLd1 and FLd3 primers, but not with FLd2 primer (data not shown). AM fungal OTU compositions of these PCR products (sequenced by the Sanger method) were similar to each other and also similar to that amplified with the eukaryote-universal primer LR1 (Supplementary Table S7). Whereas most of the sequences obtained with FLd3 were assigned to AM fungal OTUs (>88%), comparable to those obtained with LR1 (>83%), but only a half of the sequences obtained with FLd1 could be assigned to AM fungal OTUs. Accordingly, we chose FLd3, the mixture of FLd3-1, -2, -3, and -4 (Supplementary Table S1), and further assessed its performance on the MiSeq platform. The D2 region was amplified from four test samples with FLd3 and sequenced, and the reads were separated into four groups according to their 5′-primer sequence prior to OTU assignment to test whether the different primers amplify different AM fungal sequences as expected. The OTU compositions obtained with the four primers, however, were highly similar to each other (Supplementary Table S8); Bray-Curtis similarity indices between the communities obtained from the same DNA template with the different primers were consistently higher than 0.9 across all combinations (Supplementary Table S9). In addition, the numbers of the sequence read assigned to an AM fungal OTU were not markedly different among the four primers. These results suggest that mixing of the four primers differentiates the OTU compositions only marginally and that efficiencies of these primers in the amplification of D2 are not different. We therefore decided to employ FLd3-1 as a forward primer, because its sequence covers the majority of glomeromycotinan LSU rDNA.

### Soybean responses to inoculation with respect to biotic and abiotic factors

**Figure 2:**
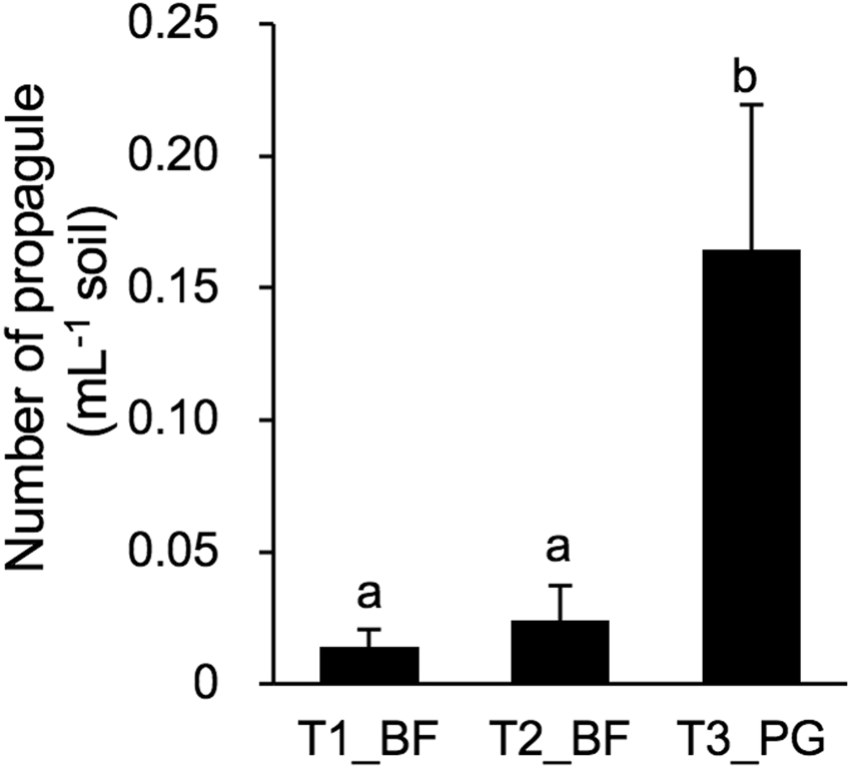
Most probable numbers (MPNs) of AM fungal propagule in the three trial sites. Soil samples were collected from four replicated plots in each trial site prior to fertilizer application and mixed with autoclaved sand to obtain a dilution series of 2^−1^, 2^−3^, 2^−5^, and 2^−7^, and *Lotus japonicus* was grown in the soil mixtures in a growth chamber for 26 days. MPNs were estimated based on the presence/absence of fungal colonization recorded under a dissecting microscope. The vertical bars indicate standard errors (*n* = 4). The different letters indicate significant differences among the three trial sites (Tukey HSD test, *p* < 0.05).

MPNs of AM fungal propagule in T1_BF and T2_BF were around 0.02 propagule mL soil^−1^, but that in T3_PG was about an order of magnitude higher (Fig. 2). Levels of available phosphate were significantly higher in T2_BF and T3_PG than in T1_BF, but P-fertilizer application did not differentiate the levels in all trials (Supplementary Table S6). Levels of exchangeable Ca and Mg were also slightly higher in T2_BF and T3_PG.

The inoculation of R-10 significantly increased shoot P concentration in the flowering stage and grain yield in T1_BF, but significantly decreased grain yield in T3_PG (Table 1). In T2_BF the inoculation did not affect shoot P concentration and grain yield. No significant effects of P-fertilizer application and its interaction with the inoculation on yield and shoot P concentration were observed in all trials. Root nodule formation was observed in all plants.

**Table 1:** Effect of AM fungal inoculation and phosphorus (P) fertilizer application on shoot P concentration of the flowering stage and grain yield of *Glycine max* in the three trials.

P application (kg ha^−1^)	Inoculum	Trial 1_Bare fallow		Trial 2_Bare fallow		Trial 3_Palisade grass	
		Shoot P (mg P g^−1^ DW)	Yield (kg ha^−1^)	Shoot P (mg P g^−1^ DW)	Yield (kg ha^−1^)	Shoot P (mg P g^−1^ DW)	Yield (kg ha^−1^)
0	Control	4.06 ± 0.09^a^	3079.9 ± 76.7	4.26 ± 0.10	3393.8 ± 41.6	4.40 ± 0.07	3192.6 ± 52.7
	R-10	4.17 ± 0.09	3273.1 ± 15.3	4.34 ± 0.09	3171.1 ± 136.4	4.36 ± 0.02	2811.6 ± 118.4
50	Control	4.10 ± 0.05	3111.1 ± 46.5	—	—	—	—
	R-10	4.20 ± 0.05	3220.4 ± 65.6	—	—	—	—
100	Control	4.06 ± 0.04	3081.4 ± 105	4.31 ± 0.09	3061.3 ± 148.7	4.60 ± 0.26	3166.3 ± 95.1
	R-10	4.30 ± 0.05	3125.5 ± 46.1	4.29 ± 0.05	3266.5 ± 83.6	4.38 ± 0.06	3103.9 ± 56.4
P		ns^b^	ns	ns	ns	ns	ns
Inoculation		**	*	ns	ns	ns	*
P × Inoculation		ns	ns	ns	ns	ns	ns

^a^±SE (*n* = 4). ^b^ANOVA: ns, not significant; *P < 0.05; **P < 0.01.

**Figure 3:**
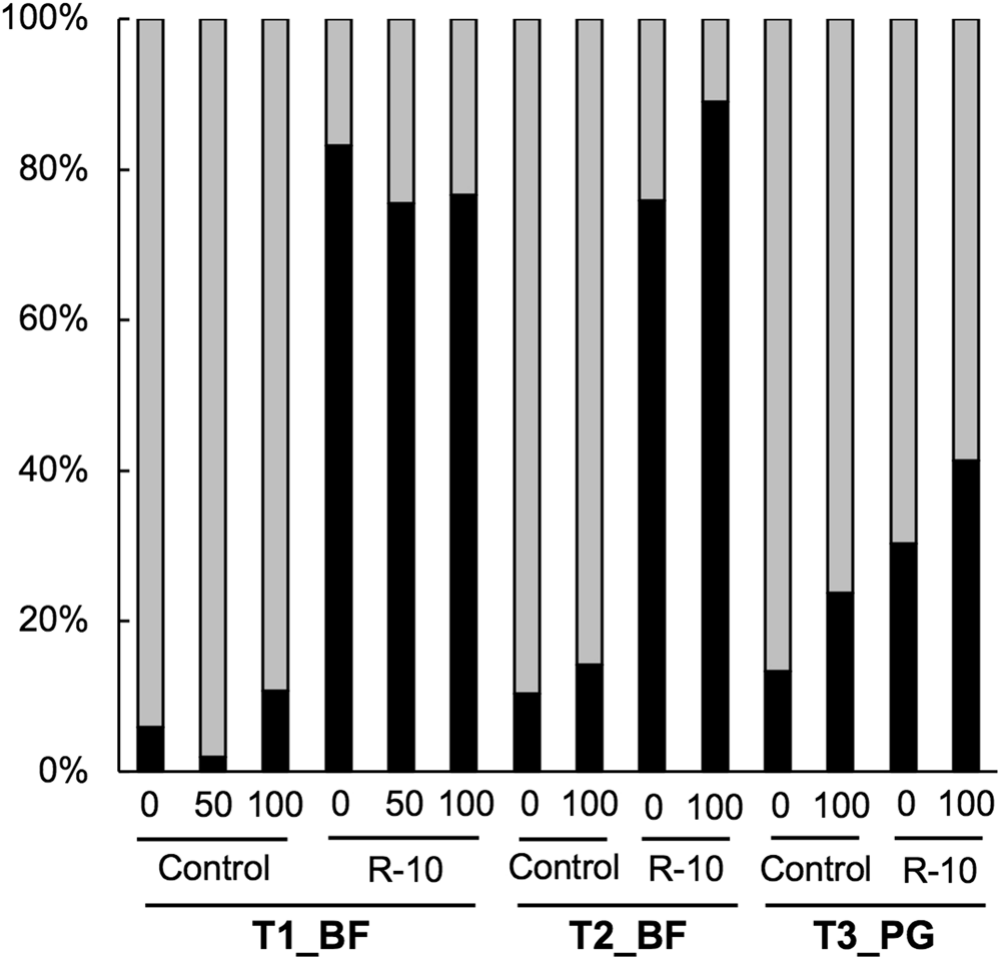
Average relative read abundance of the R-10-type operational taxonomic units (OTUs) in total AM fungal sequence read in the soybean root samples in the uninoculated (control) and inoculated (R-10) plots of the three trials (*n* = 4). The black and gray bars represent the relative read abundance of R-10-type and other OTUs, respectively.

The inoculum sequencing revealed that R-10 fungus consists of 21 OTUs (Supplementary Table S10), all of which are likely to belong to *Rhizophagus irregularis*, except for the OTU 382_Rhz that is related to *R. intraradices* (Supplementary Fig. S2). These OTUs were employed for a tracking marker in the trials. In sample sequencing, 15,000 to 30,000 high-quality reads of AM fungi were obtained for each sample and normalized to 10,000 reads per sample for community analysis. In the uninoculated control plots the R-10-type OTUs were present up to 30% of total AM fungal read (Fig. 3 and Supplementary Table S10). In T1_BF and T2_BF, however, the inoculation of R-10 drastically increased the read abundances of R-10-type OTUs to more than 70% of total read, suggesting that R-10 fungus largely dominated the communities in these trials. In contrast, the inoculation had a limited impact on the communities in T3_PG.

In multiple linear regression analysis on the relationship of environmental (biotic and abiotic) factors with the yield responses to the R-10 inoculation, the best fit model with the lowest AIC indicated that the read abundances of R-10-type OTUs is the most significant factor (*R* = 0.002, *p* < 0.001) of which a standardized partial regression coefficient was 0.67 (Table 2). In fact, the read abundances of R-10-type OTUs were positively correlated with the yield responses to the inoculation (*R*^2^ = 0.37, *p* < 0.001) (Supplementary Fig. S3), supporting the first hypothesis. In logistic regression analysis on the factors affecting the R-10 read abundances, the best fit model with the lowest AIC indicated that MPN is the most significant factor (*R* = −8.68, *p* < 0.001) of which an odds ratio was 0.00017, implying that the read abundances would be decreased by 0.00017-fold with increasing MPN by 1 (Table 3). In fact, the read abundances of R-10-type OTUs in the inoculated plots were negatively correlated with the MPNs (*R* = −0.73, *p* < 0.001) (Supplementary Fig. S4), supporting the second hypothesis.

**Table 2:** Effect size of biotic and abiotic factors on yield response to R-10 inoculation estimated by multiple linear regression analysis.

Variable	Coefficient estimate	Standard error	*P* value	Standardized partial regression coefficient
Read abundance of R-10-type OTUs	0.00205	0.00046	0.00019	0.67
P application level	0.00033	0.00023	0.16	0.21
Phosphate absorption coefficient	0.00013	0.00008	0.12	0.24

**Table 3:** Effect size of biotic and abiotic factors on the read abundance of R-10-type OTUs estimated by logistic regression with a random effect model.

Variable	Coefficient estimate	Standard error	*P* value^a^	Odds ratio
P application level	0.0041	0.0013	0.0013	1.00
Phosphate absorption coefficient	−0.0019	0.00091	0.037	1.00
MPN^b^	−8.68	2.01	1.5.E-05	0.00017
Nitrate nitrogen	0.63	0.30	0.035	1.87
Exchangeable potassium	−0.097	0.04	0.023	0.91

^a^Neyman-Pearson test.

^b^Most probable numbers of AM fungal propagule.

### Interactions between introduced and indigenous AM fungi

**Figure 4:**
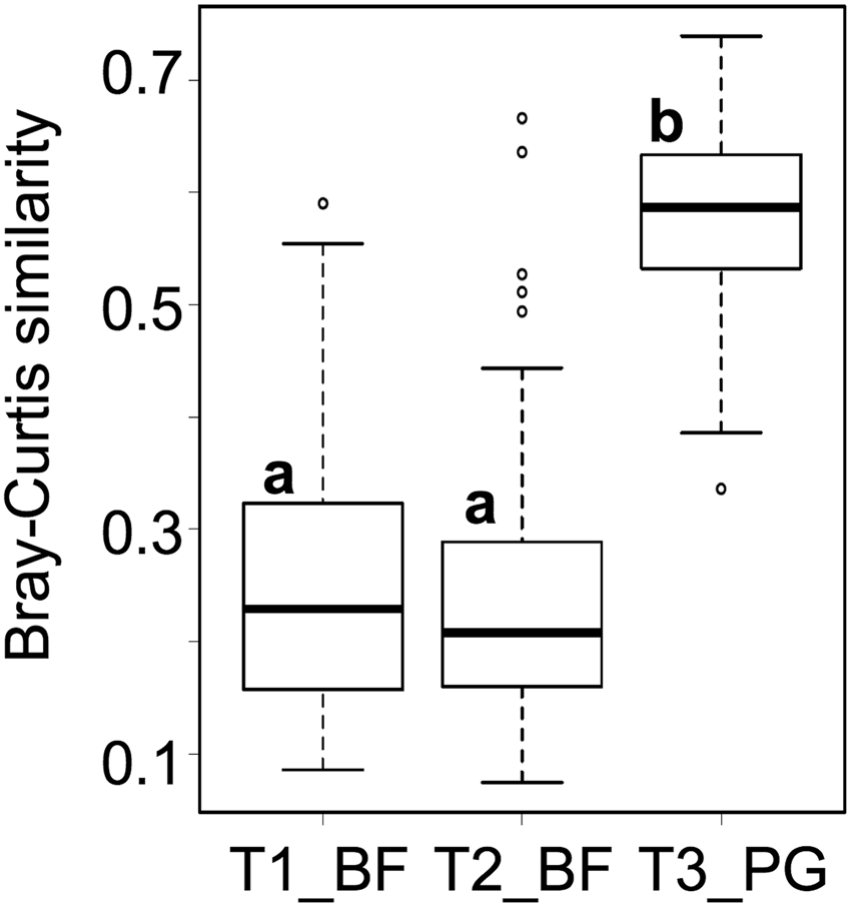
Box plots of Bray-Curtis similarity index between the inoculated and uninoculated communities in the three trials. The bottom, top, and median lines of the boxes represent the first quantiles, third quantiles, and medians, respectively, and the error bars represent the range of the data. The different letters indicate significant differences among the three trials (Steel-Dwass test, *p* < 0.05).

Interactions between the inoculum and indigenous AM fungi were first dissected via assessing the impact of inoculation on the community compositions. Two-way PERMANOVA indicated that the community compositions were different among the three trial fields and that the inoculation, as well as the interaction, had a significant impact on the compositions (Supplementary Table S11). Bray-Curtis similarity index between the inoculated and control plots was significantly higher in T3_PG than in T1_BF and T2_BF (Fig. 4). These results imply that the impact of R-10 inoculation on the community compositions was smaller in T3_PG; that is, the indigenous communities in T3_PG were more robust against the inoculation than those in T1_BF and T2_BF.

**Figure 5:**
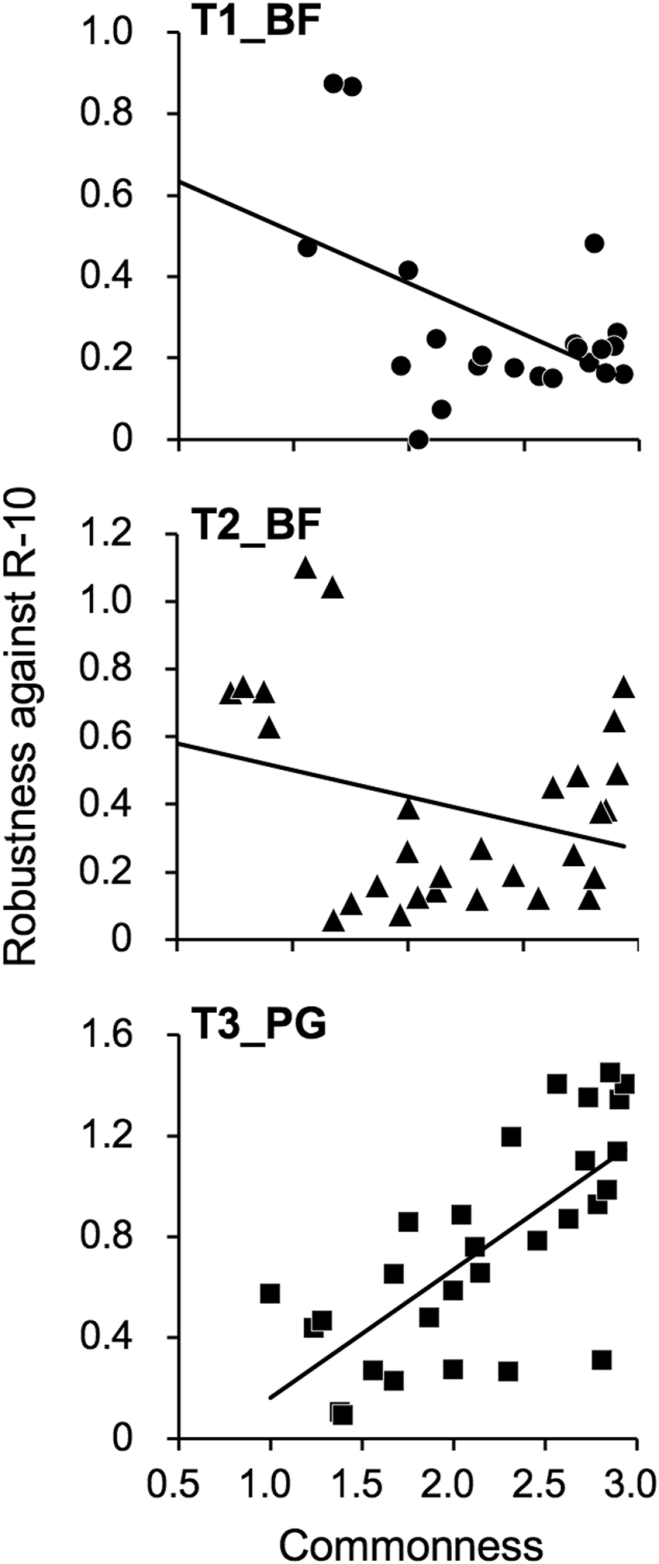
Correlation analysis between commonnesses and robustnesses of indigenous fungal OTUs in T1_BF (circles), T2_BF (triangles), and T3_PG (squares). Commonness that was referred to as niche breadth was calculated according to Pandit *et al*.^45^ to evaluate their habitat specialization, and robustness against the inoculation of R-10 fungus was calculated as a ratio of the mean read abundance in the inoculated plots to that in the control plots. Pearson correlation coefficients (*R*) were −0.50 (*p* = 0.018), −0.29 (*p* = 0.12), and 0.72 (*p* < 0.001) in T1_BF, T2_BF, and T3_PG, respectively.

To characterize the indigenous fungi that compete with R-10 fungus, the two indices commonness and robustness against the inoculation were calculated for each indigenous fungal OTU (Supplementary Table S12), except for the R-10-type OTUs, because the OTUs originate from R-10 fungus could not be discriminated from those present in the indigenous communities. The values of commonness were rather negatively correlated with those of robustness in T1_BF (*R* = −0.50, *p* = 0.018) and T2_BF (*R* = −0.29, *p* = 0.12), implying that more common fungi were generally less robust against the introduction of R-10 fungus in these trial sites (Fig. 5). In contrast, there was a positive correlation between commonness and robustness in T3_PG (*R* = 0.72, *p* < 0.001); more common fungi were more robust against the introduction of R-10 fungus. These results imply that R-10 fungus competed for the niche mainly with the fungi that are commonly distributed in the trial sites.

## Discussion

It has been proposed that abundance of indigenous AM fungi^18,46^, soil fertility^4^, and crop rotation^16^, as well as plant and fungal genotypes^47,48^, are the major factors that determine inoculation success. In these studies, however, the colonization of inoculum fungi could not be taken into account due to technical difficulties. In the present study, not only the environmental factors but also the interactions between the inoculum and indigenous fungi were incorporated in the modeling of the soybean yield responses to AM fungal inoculation, which provides a new framework for evaluating the significance of environmental factors towards successful application of the fungi in agricultural production.

Recently, technical feasibility for tracking AM fungal inocula in field trials has been demonstrated^2,19,23,49^, but methods that enable to analyze interactions between introduced and indigenous fungi on high-throughput sequencing platforms have not yet been proposed. In this study, no inoculum-specific OTUs, that is, those that were absent in the field, but abundant in the inoculum fungus, were found, which implies that increases in the inoculum OTUs should be interpreted carefully. In the inoculated plots of T1_BF and T2_BF, the drastic increases in the read abundance of R-10-type OTUs was observed, implying that the inoculum fungus was highly likely to dominate in these plots. It was not possible, however, to estimate what proportion of the sequence reads was of the inoculum fungus. Accordingly, it is considered that the present method could be applicable only if (i) inoculum fungi have specific OTUs or (ii) dramatic increases in inoculum fungal OTUs by inoculation are observed in the presence of the same OTUs in indigenous community.

Our modeling indicated that the read abundances of inoculum OTUs in the flowering stage is the most important factor that determine the soybean yield responses to the inoculation. These observations strengthen the findings in the meta-analysis^50^ that demonstrated that rapid colonization and dominance of inoculum fungi in early growth stages are essential to obtain positive yield responses to AM fungal inoculation. In the case of soybean, it seems also likely that the positive yield responses were not only due to the direct effect of the inoculation but also due to an indirect effect of the inoculation, such as enhancement of rhizobial N fixation through improving P nutrition. It has been well documented that N fixation in the nodules is largely limited by P nutrition even under conditions of adequate P fertilization (reviewed in Graham and Vance^51^). This idea further raises the following two questions; (i) whether the effectiveness of AM fungal inoculation could be enhanced under conditions of abundant rhizobia and (ii) whether AM fungal inoculation could reduce N fertilizer input via enhancing N fixation without reduction in yield. Although levels of nodule formation (e.g., nodule number and weight) were not evaluated in the present study, it would be worthwhile to incorporate these factors for the modeling.

Unexpectedly, increases in shoot P concentration and grain yield were not observed in the inoculated plots of T2_BF in which the large dominance of R-10-type OTUs was observed. These observations suggest that read abundances of inoculum fungal OTUs would not be a universal indicator to predict the effectiveness of inoculation and, further, that more comprehensive analysis on environmental factors, together with the read abundances of inoculum OTUs, is necessary. In T2_BF it seems likely that the high levels of available phosphate in the soil cancelled the benefit of inoculation (e.g., Tawaraya *et al*.^52^; Verbruggen *et al*.^4^), although the multiple linear regression analysis indicated that available phosphate was not a significant factor for the responsiveness of soybean. More field trials in a wider range of environments are required to improve reliability of the modeling.

The levels of the colonization (i.e. read abundances) of inoculum fungus were negatively correlated with the propagule density of indigenous AM fungi, although these data were obtained from only three trials. It has been suggested that indigenous AM fungal population can be maintained by cropping of AM plants, but is decreased by non-host cropping^53^ and bare fallow^18,46^. In addition, the growth/yield gap after bare fallow could be recovered by the reintroduction^46^ or inoculation^18^ of AM fungi. Our results support these findings via analyzing the interactions between the introduced and indigenous fungi. Further trials, however, should be conducted in arable fields with various agricultural practices/management in diverse environments to confirm the significance of propagule density in the colonization of introduced fungi.

In agricultural ecosystems frequent disturbance (e.g., tillage) may act as selection pressure for the fungi that have the life history strategy of ‘ruderal’^54^, that is, those that colonize rapidly^55^, regenerate hyphal networks efficiently after disturbance^56^, and produce abundant spores^57^. According to this concept, the inoculum fungus *R. irregularis* (R-10) might have typical ruderal traits; it colonizes roots in early growth stages as observed in this study, produces abundant spores^3^, and is well adapted to agricultural ecosystems (Peyret-Guzzon *et al*.^58^), which would be the essential traits for agriculture application. On the other hand, given that the indigenous fungi that competed with R-10 fungus were those that showed a higher value of commonness, their life history strategy is the same as that of R-10 fungus; they might also be ruderal. It is considered that these indigenous ruderals might largely proliferate during the cropping of palisade grass in the previous year 2014, resulted in the increases in MPN in T3_PG, because our protocol for the assessment of MPN would detect mainly the propagule that is capable of colonizing rapidly (i.e. within 26 days, Supplementary Methods S1). Therefore, population size of indigenous ruderal fungi, which could be estimated by MPN, is likely to be a key to predict the level of the colonization of inoculum fungus in the field.

Introduction of excess AM fungal propagule to the soil with high potential of indigenous AM fungi may decrease plant growth responses to the fungi. Janoušková *et al*.^59^ demonstrated that AM fungal inoculation, particularly at high rates of infective propagule, decreased host growth responses to the inoculation in the presence of a preestablished synthetic community in a model experiment. They suggested that excess propagule might enhance competition among the fungi for host resources, which consequently decreased the benefit of AM symbiosis. Our results that grain yield was decreased by the inoculation in T3_PG in which propagule density was higher than the others support their findings at the field level.

In this study, a new primer FLd3 was first designed as a mixture of the four different oligo DNAs in the conserved region between the D1 and D2 in the LSU rDNA. It was unexpected, however, that the OTU compositions obtained with each of the oligo DNAs were highly similar to each other. Basically, FLd3 was designed by taking into account the sequence variations in the region within the Glomeromycotina, because sequence mismatches generally bias the compositions in amplicon-based studies^60^. It seems likely that the annealing temperature (48 °C) in the thermal cycling program, which was determined according to the melting temperature (*T*m) of the reverse primer FLR2, was actually 6–8 °C lower than the *T*m of the four oligo DNAs and thus might allow them to anneal to the priming site even in the presence of one or two mismatches/gaps. The fact that the OTU composition obtained with the eukaryote-universal primer LR1 was also highly similar to that obtained with FLd3 further indicates the validity of this primer; it is capable of amplifying a wide range of glomeromycotinan fungi with a minimum bias as confirmed with LR1 primer^11,28,30^.

## Conclusion

Our modeling demonstrated that the dominance of inoculum fungus in the flowering stages is a necessary condition to obtain positive yield responses to the inoculation, which would be largely determined by agricultural practices/management, e.g., via altering the propagule density of indigenous AM fungi. In addition to agricultural practices/management, plant genotypes, which were not tested in this study, also critically affect growth responses to AM fungal inoculation (e.g., Sawers *et al*.^48^) and thus could be an important factor. More field trials covering a wide range of plant cultivars in diverse environments are necessary to establish a more practical prediction model. The analysis on niche competition between the introduced and indigenous fungi suggests that ruderal fungi play a key role in conventional (intensive) agriculture, which raises a question of how significant the ruderal traits are in less-intensive agriculture, such as no-till management and organic agriculture. Verbruggen *et al*.^10^ observed that species richness of AM fungi declines progressively with increasing management intensity. This observation leads to the idea that the fungi in less-intensive agricultural fields would be more diverse not only taxonomically but also functionally; for example, the ‘competitor’ fungi that proliferate mainly through hyphal networks^54^ may dominate in no (or reduced)-till fields instead of ruderal fungi. It is of interest to characterize AM fungal communities in less intensive agricultural fields through introduction of a ruderal fungal inoculum.

## Electronic supplementary material

Supplementary information

Dataset
