# Supplementary material for: Dissection of niche competition between introduced and indigenous arbuscular mycorrhizal fungi with respect to soybean yield responses
Source: Sci Rep. 2018 May 9;8:7419. doi: 10.1038/s41598-018-25701-4 (PMC5943277; doi:10.1038/s41598-018-25701-4)
Supplement: Supplementary file 1 — Supplementary information [file 41598_2018_25701_MOESM1_ESM.pdf]

Supplementary information

Dissection of niche competition between introduced and indigenous arbuscular mycorrhizal fungi with respect to soybean yield responses

Rieko Niwa[1*], Takuya Koyama[2*], Takumi Sato[3], Katsuki Adachi[2], Keitaro Tawaraya[3], Shusei Sato[4,5], Hideki Hirakawa[5], Shigenobu Yoshida[1] and Tatsuhiro Ezawa[6†]

* These authors contributed equally to this work.

[1]Central Region Agricultural Research Center, National Agriculture and Food Research Organization (NARO), 2-1-18 Kannondai, Tsukuba 305-8666, Japan

[2]Kyushu Okinawa Agricultural Research Center, NARO, 6651-2 Miyakonojo, Miyazaki 885-0091, Japan

[3]Faculty of Agriculture, Yamagata University, Tsuruoka 997-8555, Japan

[4]Graduate School of Life Sciences, Tohoku University, Sendai 980-8577, Japan

[5]Kazusa DNA Research Institute, Kisarazu 292-0818, Japan

[6]Graduate School of Agriculture, Hokkaido University, Sapporo 060-8589, Japan

[†]Author for correspondence:
Tatsuhiro Ezawa
Tel: +81 11 857 9732
Email: tatsu@res.agr.hokudai.ac.jp

Supplementary Methods S1. Most probable number of AM fungal propagule

The soil samples were dried in a greenhouse, passed through a 2-mm stainless sieve, and mixed with autoclaved sand to obtain a dilution series of 2^{-1}, 2^{-3}, 2^{-5}, and 2^{-7}. For R-10 inoculum, the same dilution series were prepared with autoclaved sand. Pre-germinated seeds of *Lotus japonicus* cv. Miyakojima were transplanted to the mixtures in 50 mL plastic tubes and grown in a growth chamber at 25°C in a 16-h photoperiod for 26 days (n = 4), and then the roots were harvested, washed with tap water, cleared in 10% KOH at 80°C for 3 h, acidified by 2% HCl, and stained with 0.05% (w:v) trypan blue in lactoglycerol at 90°C for 15 min. The presence and absence of fungal colonization was recorded under a dissecting microscope, and the most probable number of AM fungal propagule was estimated according to Cochran (1950)[1].

Supplementary Methods S2. Site description

The site is located in the subtropical zone; the hourly maximum and minimum temperatures from July to Nov were 35.3˚C on 31 July and 2 Aug and 4.3˚C on 27 Nov, respectively, and the average temperature during the period was 19.1˚C in 2015. The annual rainfall was 3326 mm, one third of which was recorded in June in 2015. These climatic data were obtained at Miyakonojo observatory, Japan Meteorological Agency, which is located approximately 9 km from the experimental field. The soil of the fields belongs to the order Andosol that is loam in texture, volcanic ash, deep percolating, and well drained.

Supplementary Methods S3. Primer design and validation

We conducted *in silico* analysis of published sequences of 28 cultured and 84 uncultured species/taxons (112 sequences in total) in the Glomeromycotina to design PCR primers for the D2 (Supplementary Table S2). Three forward primers, FLd1, FLd2, and FLd3, were designed in the conserved region between the D1 and D2 (Figure 1 and Supplementary Table S1). FLd1, FLd2, and FLd3 are mixtures of 3, 6, and 4 different oligo DNAs, respectively, and cover 94.6, 95.5 and 93.8%, respectively, of species/taxons in the 112 sequences (Supplementary Table S3). To evaluate performance of these

primers by comparing with the eukaryote-universal forward primer LR1[2], the region was amplified in combination with the fungi-specific reverse primer FLR2[3] using a DNA template of a maize test sample (roots) collected from the experimental field in Hokkaido University in 2014. The reaction mixtures consisted of Expand High-Fidelity PLUS PCR System (Roche Diagnostics, Tokyo), 0.5 nmol μL^{-1} of the forward and reverse primers, and 1.0-μL DNA template in a total of 25 μL, and C1000 Touch Thermal Cycler (BIO-RAD, Tokyo) was employed for amplification. The thermal cycling program was as follows: initial denaturation at 94 ˚C for 2 min, followed by 30 cycles of denaturation at 94 ˚C for 15 s, annealing at 48 (FLd1), 44 (FLd2), 48 (FLd3), or 50 (LR1) ˚C, polymerization at 72 ˚C for 1 min, and final extension at 72 ˚C for 10 min. The PCR products were cloned into pT7Blue T-vector (Merch, Tokyo) according to the manufacturer's instructions, and nucleotide sequences of 50 clones randomly chosen from each sample (library) were determined using BigDye Terminator v3.1 Cycle Sequencing Kit with ABI PRISM 3130*xl* Genetic Analyzer (Applied Biosystems, Tokyo).

Given that the new primers were mixtures of different oligo DNAs, more detailed analysis on the community compositions obtained by a selected primer set, the D2 was amplified using DNA templates obtained from four test samples, two soybean samples grown in Tsukuba and Tsugaru and two maize samples grown in Nakashibetsu and Morioka (Supplementary Table S4), under the same conditions and sequenced on the MiSeq platform.

Supplementary Methods S4. Database construction

Quarter million sequences of AM fungal D2 region obtained by Sanger[4-7] and Roche 454[8] sequencing were clustered together with the published sequences at ≥ 95% sequence similarity with CD-HIT program[9], and 224 representative sequences were selected from each cluster (we preferentially selected those obtained from cultured species, if possible) as operational taxonomic units (OTUs). In addition, 4,750 glomeromycotinan sequences were obtained from Ribosomal Database Project[10] and queried against the 224 OTUs with the BLASTN program (2.2.29+)[11], and those that did not showed similarity to any of the OTUs at ≥ 95% (1,511 sequences) were clustered at ≥ 95% similarity using CD-HIT[9]. From each of the clusters, 116 representative sequences were selected and combined with

the 224 OTUs, resulted in a total of 340 OTUs. For further enrichment of the database, we sequenced a total of 824 PCR products from soybean, maize, and Welsh onion roots grown throughout Japan (Supplementary Table S4) on the MiSeq platform and obtained a total of 30 million sequence reads in which 1,167,844 reads (i.e. approx. 4% of total read) could not be assigned to any of the 340 OTUs in the database. These unassigned reads were first clustered at 100% similarity, and 175 clusters that consisted of at least 0.05% of total reads were selected and further clustered at ≥ 95% similarity. Then, representative sequences were selected from each cluster, aligned, and analyzed phylogenetically according to the four criteria described below, and 72 OTUs were newly defined in addition to the 340 OTUs (412 OTUs in total). All sequences of the OTUs met the following criteria: (1) the lengths are more than 330 bp, including the primer regions, (2) they are not chimeric sequences, which was carefully assessed by comparing published sequences, and (3) have no undetermined nucleotide (i.e. 'n'), and (4) they are assigned to one of the major clades of Glomeromycotina, which was analyzed both by phylogenetic analysis (Supplementary Methods S5) and by BLASTN searches against GenBank database. For assignment of non-glomeromycotinan sequences, fungal LSU rDNA sequences, except for those of glomeromycotinan fungi, were obtained from Ribosomal Database Project[10], clustered at 100% sequence identity. The resultant *c.* 80,000 sequences were combined with the AM fungal OTU database.

Supplementary Methods S5. Phylogenetic analysis

A total of 412 sequences of the AM fungal OTUs were aligned together with the published AM fungal sequences selected across all families (reference sequences) using MAFFT version 7[12], and a maximum likelihood phylogenetic analysis was computed with RAxML version 8[13] using 1000 bootstraps and GTRGAMMA model for both bootstrapping and tree inference.

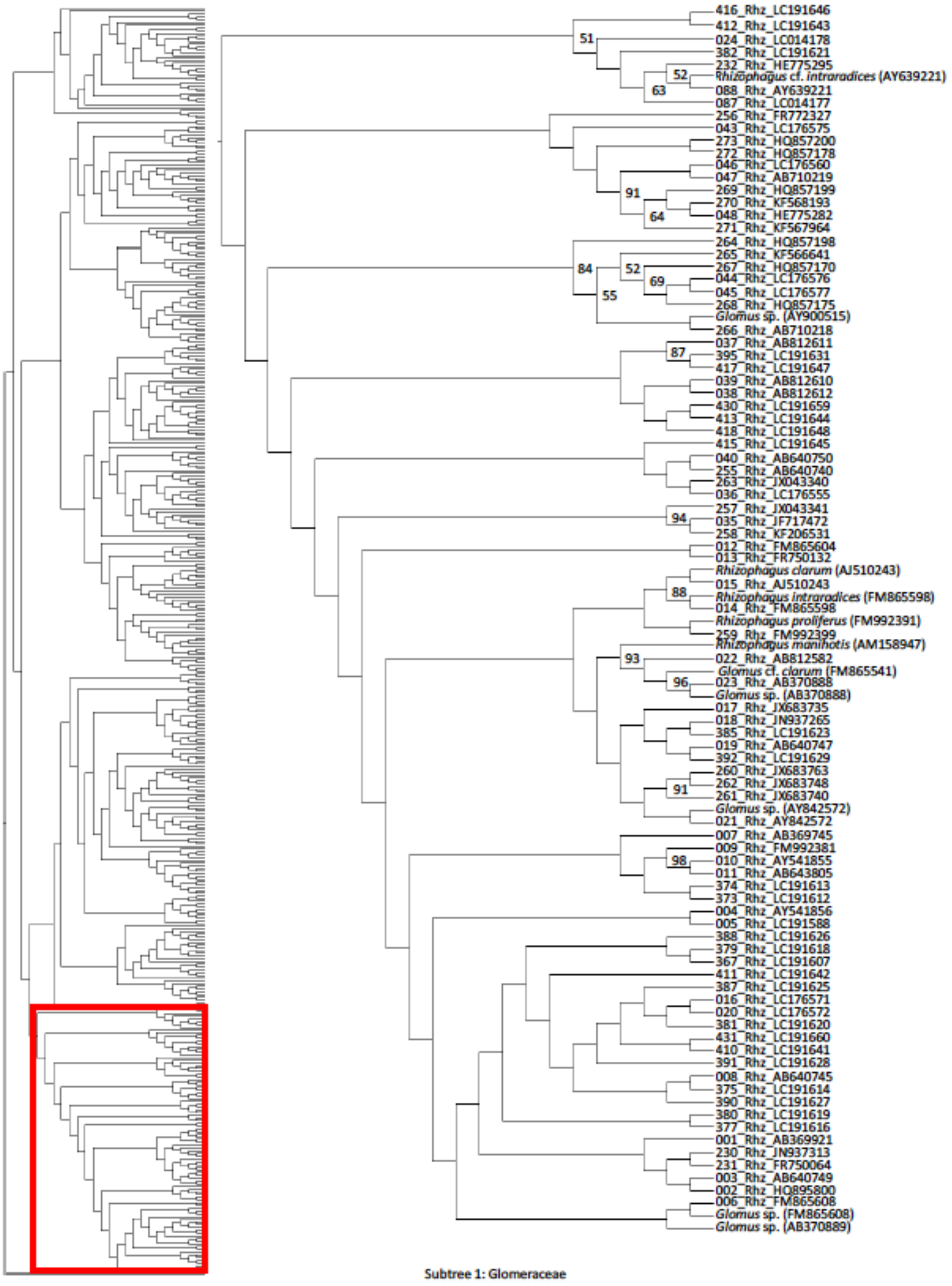

Supplementary Figure S1. Maximum likelihood phylogenetic tree of 412 operational taxonomic units (OTUs) of arbuscular mycorrhizal fungi in the database based on the partial LSU rDNA sequences. Bootstrap values more than 50% are indicated. Subtree 1 – 3: Glomeraceae; Subtree 4: Pacisporaceae and Gigasporaceae; Subtree 5: Diversisporaceae and Acaulosporaceae; Subtree 6: Paraglomeraceae, Archaerosporaceae, Ambisporaceae, and uncultured Glomeromycotina; Subtree 7: Claroideoglomeraceae.

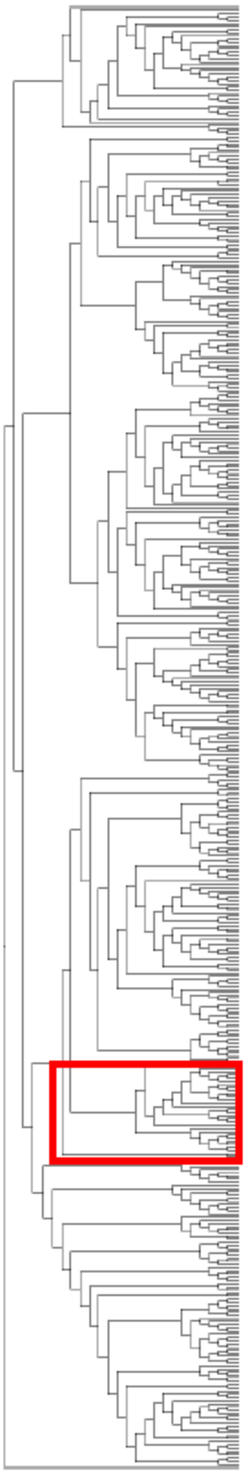

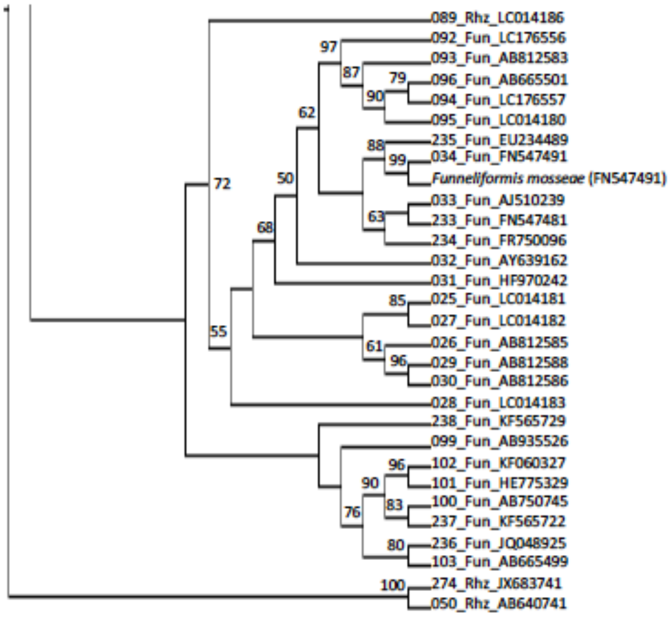

Subtree 2: Glomeraceae (*continued*)

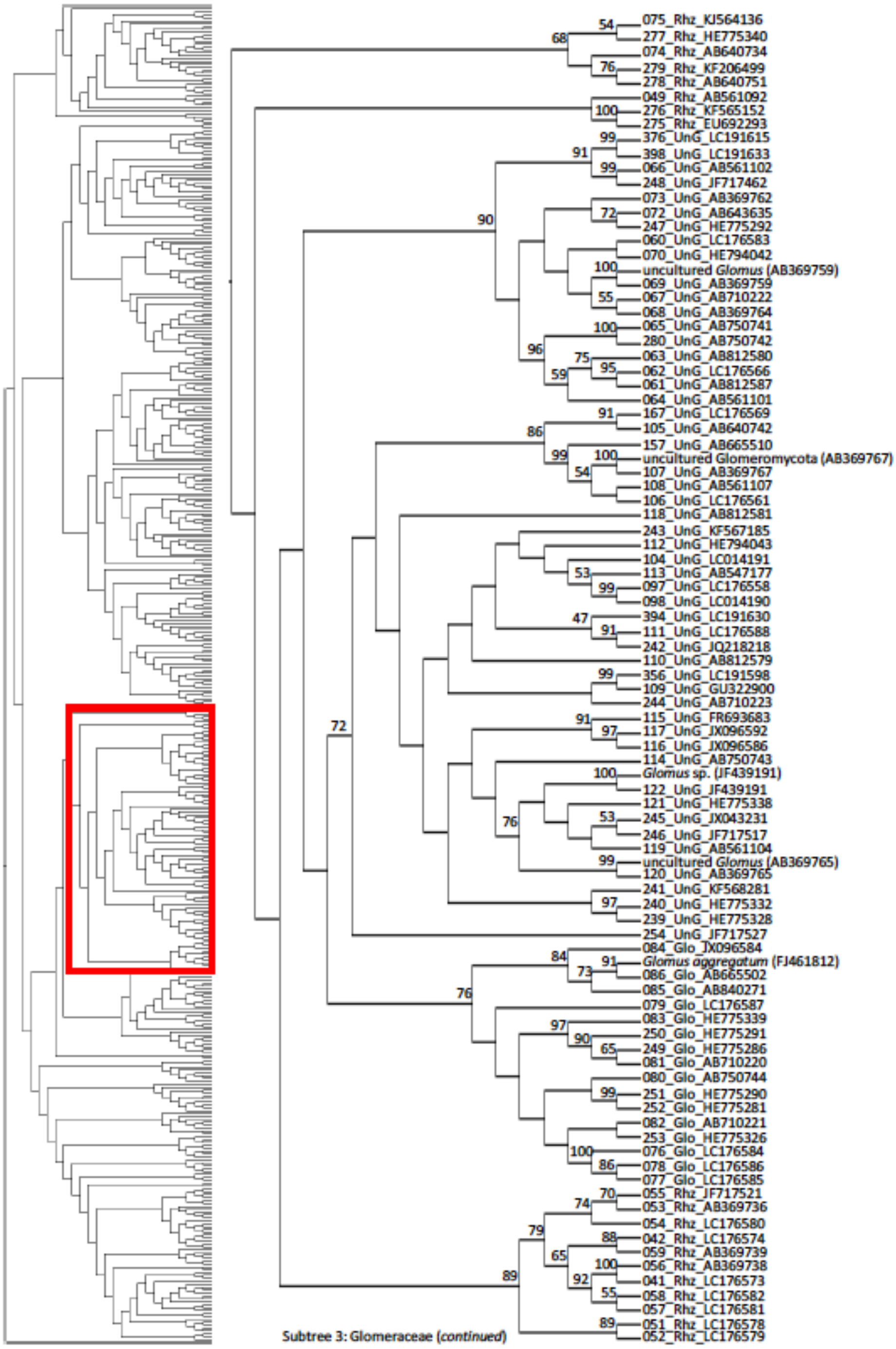

Subtree 3: Glomeraceae (*continued*)

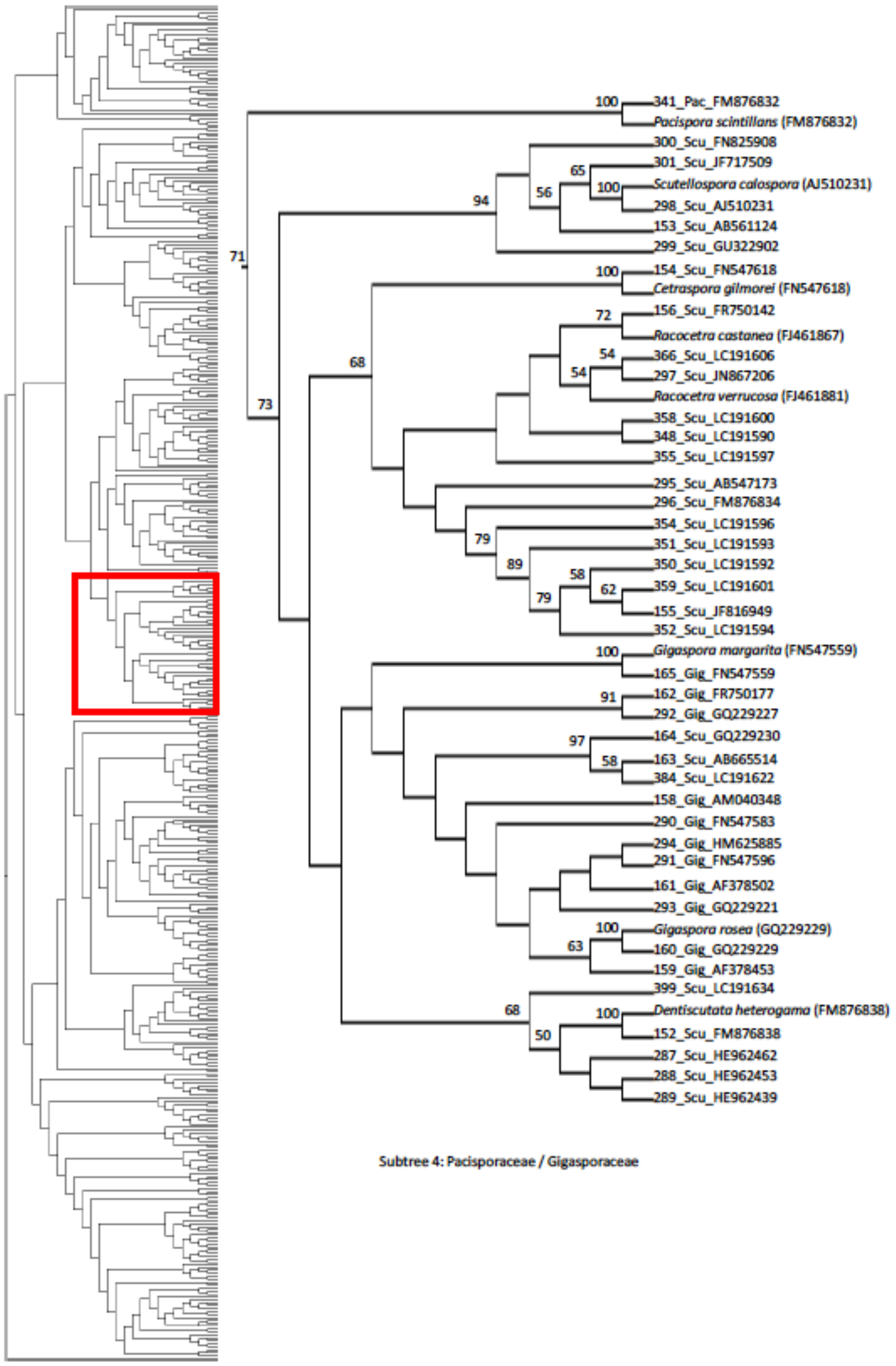

Subtree 4: Pacisporaceae / Gigasporaceae

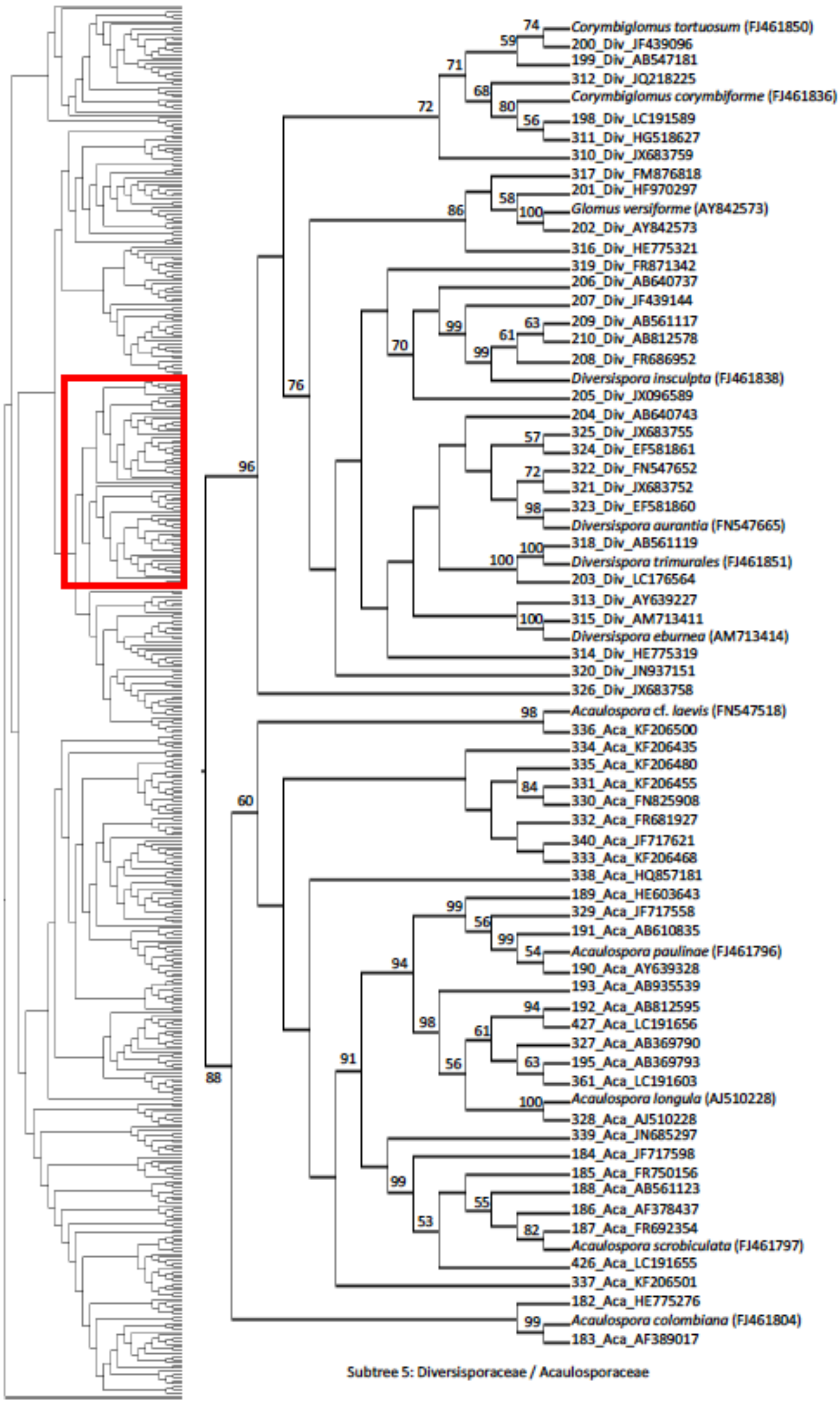

Subtree 5: Diversisporaceae / Acaulosporaceae

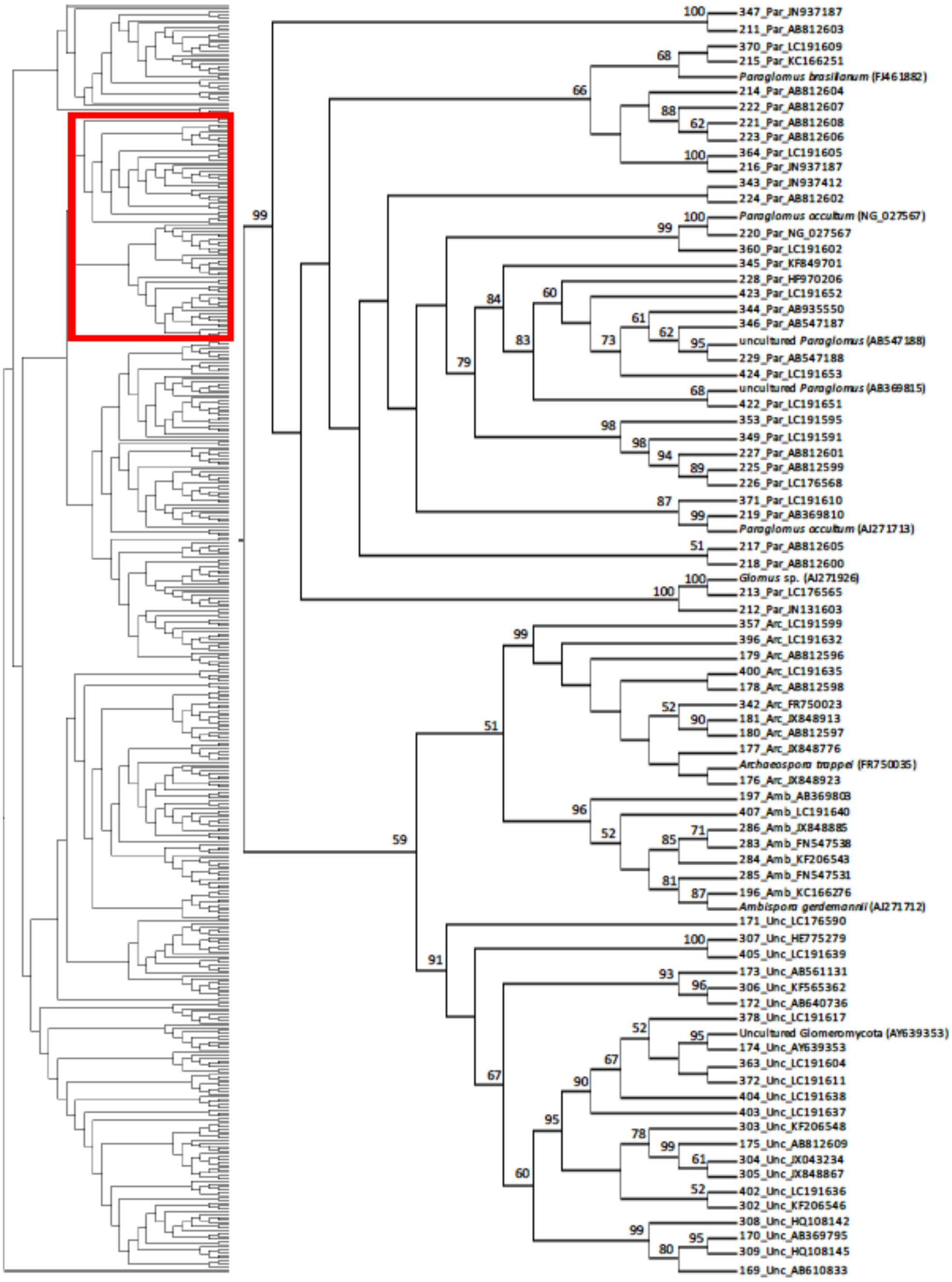

Subtree 6: Paraglomeraceae / Archaerosporaceae / Ambisporaceae / Uncultured Glomeromycotina

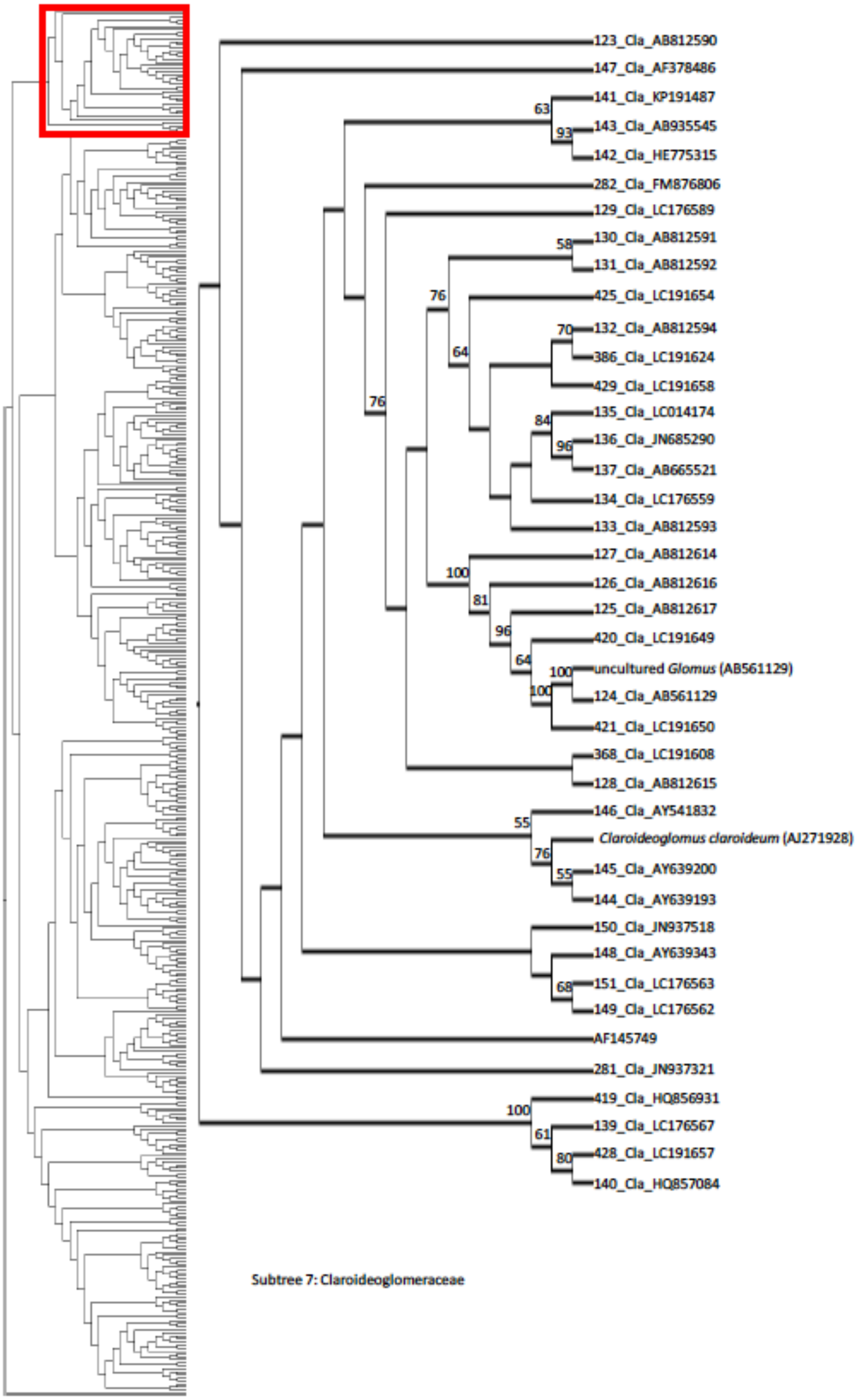

Subtree 7: Claroideoglomeraceae

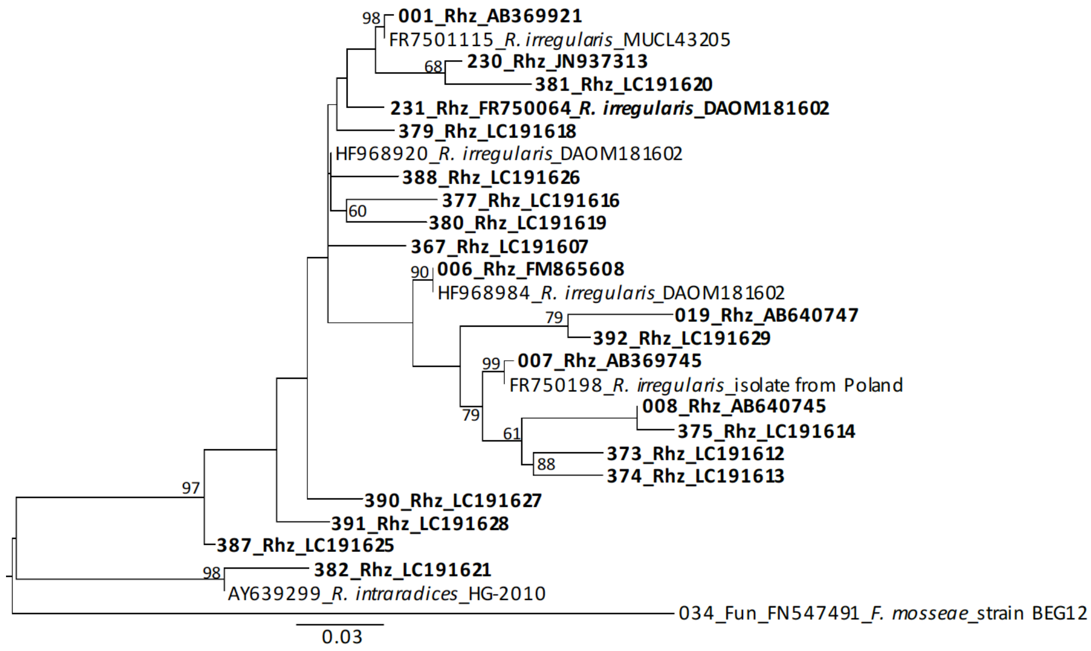

Supplementary Figure S2. Maximum likelihood phylogenetic tree of R-10-type operational taxonomic units (OTUs, bold letters) with the published sequences of *Rhizophagus irregularis* (FR750115, FR750198, HF968920, and HF968984) and *R. intraradices* (AY639299) based on the partial LSU rDNA sequences. Bootstrap values more than 60% are indicated. The OTU 034_Fun (*Funneliformis mosseae* BEG12, FN547491) was employed as the outgroup.

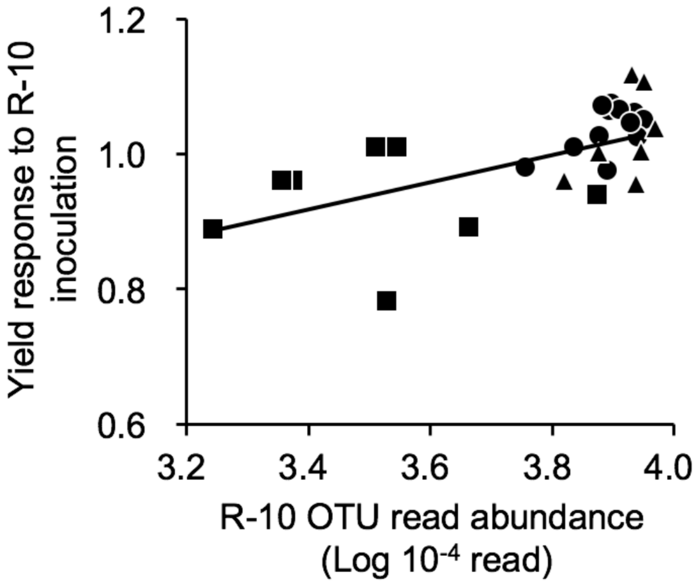

Supplementary Figure S3. Correlation between read abundances of the R-10-type OTUs and soybean yield responses to the inoculation of R-10 fungus. Yield response to the inoculation were calculated by dividing the yield in the inoculated plot by the mean yield in the control plots and plotted against the read abundances of R-10-type OTUs (log-transformed) in the inoculated plots of T1_BF (circles), T2_BF (triangles), and T3_PG (squares). Pearson correlation coefficient (R) = 0.61, $p < 0.001$ ($n = 27$).

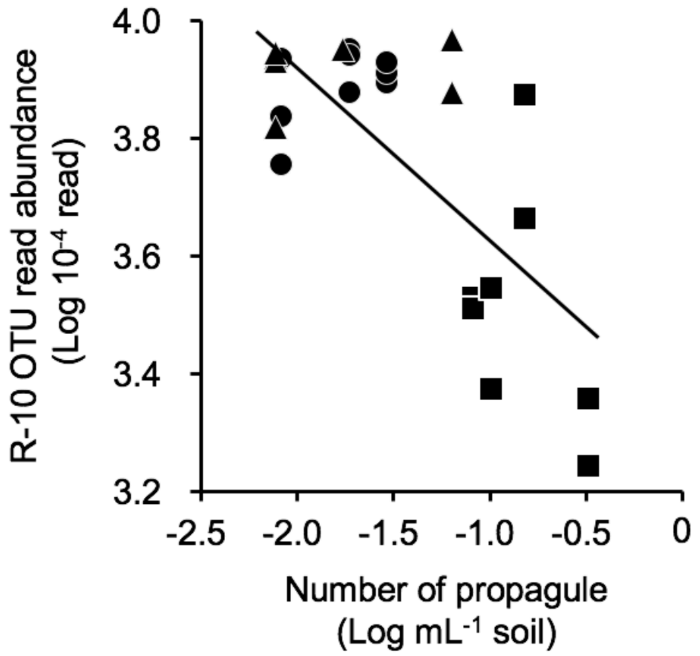

Supplementary Figure S4. Correlation between most probable numbers (MPNs) of AM fungal propagule and read abundances of the R-10-type OTUs. Relative read abundances of the R-10-type OTUs (log-transformed number per 10^4 reads) in the inoculated plots of T1_BF (circles), T2_BF (triangles), and T3_PG (squares) were plotted against MPNs (log-transformed number). Pearson correlation coefficient (R) = –0.73, $p < 0.001$ (n = 24).

References

1 Cochran, W. G. Estimation of bacterial densities by means of the "most probable number". *Biometrics* **6**, 105-116, doi:10.2307/3001491 (1950).

2 van Tuinen, D., Jacquot, E., Zhao, B., Gollotte, A. & Gianinazzi-Pearson, V. Characterization of root colonization profiles by a microcosm community of arbuscular mycorrhizal fungi using 25S rDNA-targeted nested PCR. *Mol Ecol* **7**, 879-887, doi:10.1046/j.1365-294x.1998.00410.x (1998).

3 Trouvelot, S., van Tuinen, D., Hijri, M. & Gianinazzi-Pearson, V. Visualization of ribosomal DNA loci in spore interphasic nuclei of glomalean fungi by fluorescence in situ hybridization. *Mycorrhiza* **8**, 203-206, doi:10.1007/s005720050235 (1999).

4 Cheng, Y., Ishimoto, K., Kuriyama, Y., Osaki, M. & Ezawa, T. Ninety-year-, but not single, application of phosphorus fertilizer has a major impact on arbuscular mycorrhizal fungal communities. *Plant Soil* **365**, 397-407, doi:10.1007/s11104-012-1398-x (2013).

5 Kawahara, A., An, G. H., Miyakawa, S., Sonoda, J. & Ezawa, T. Nestedness in arbuscular mycorrhizal fungal communities along soil pH gradients in early primary succession: acid-tolerant fungi are pH generalists. *Plos One* **11**, doi:10.1371/journal.pone.0165035 (2016).

6 An, G. H., Miyakawa, S., Kawahara, A., Osaki, M. & Ezawa, T. Community structure of arbuscular mycorrhizal fungi associated with pioneer grass species *Miscanthus sinensis* in acid sulfate soils: Habitat segregation along pH gradients. *Soil Sci Plant Nutrit* **54**, 517-528, doi:10.1111/j.1747-0765.2008.00267.x (2008).

7 Kawahara, A. & Ezawa, T. Characterization of arbuscular mycorrhizal fungal communities with respect to zonal vegetation in a coastal dune ecosystem. *Oecologia* **173**, 533-543, doi:10.1007/s00442-013-2622-y (2013).

8 Yoneyama, K. *et al.* Difference in Striga-susceptibility is reflected in strigolactone secretion profile, but not in compatibility and host preference in arbuscular mycorrhizal symbiosis in two maize cultivars. *New Phytol.* **206**, 983-989, doi:10.1111/nph.13375 (2015).

9 Fu, L. M., Niu, B. F., Zhu, Z. W., Wu, S. T. & Li, W. Z. CD-HIT: accelerated for clustering the next-generation sequencing data. *Bioinformatics* **28**, 3150-3152, doi:10.1093/bioinformatics/bts565 (2012).

10 Cole, J. R. *et al.* Ribosomal Database Project: data and tools for high throughput rRNA analysis. *Nucleic Acids Res* **42**, D633-D642, doi:10.1093/nar/gkt1244 (2014).

11 Camacho, C. *et al.* BLAST plus : architecture and applications. *BMC Bioinformatics* **10**, doi:10.1186/1471-2105-10-421 (2009).

12 Katoh, K. & Toh, H. Recent developments in the MAFFT multiple sequence alignment program. *Brief Bioinform* **9**, 286-298, doi:10.1093/bib/bbn013 (2008).

13 Stamatakis, A. RAxML version 8: a tool for phylogenetic analysis and post-analysis of large phylogenies. *Bioinformatics* **30**, 1312-1313, doi:10.1093/bioinformatics/btu033 (2014).
